# Computerised cognitive behavioural therapy for the treatment of depression in people with multiple sclerosis: external pilot trial

**DOI:** 10.1186/1745-6215-12-259

**Published:** 2011-12-14

**Authors:** Cindy L Cooper, Daniel Hind, Glenys D Parry, Claire L Isaac, Munyaradzi Dimairo, Alicia O'Cathain, Anita Rose, Jennifer V Freeman, Leonie Martin, Eva C Kaltenthaler, Anna Thake, Basil Sharrack

**Affiliations:** 1 Clinical Trials Research Unit, ScHARR, University of Sheffield, Regent Court, 30 Regent Street, Sheffield, S1 4DA, UK; 2 ScHARR, University of Sheffield, Regent Court, 30 Regent Street, Sheffield, S1 4DA, UK; 3 Clinical Psychology Unit, Department of Psychology, The University of Sheffield, Western Bank, Sheffield S10 2TP, UK; 4 The Walton Centre of Neurology & Neurosurgery, Liverpool L9 7LJ, UK; 5 Department of Neurology, Royal Hallamshire Hospital, Sheffield S10 2JF, UK

## Abstract

**Background:**

People with multiple sclerosis (MS) are at high risk of depression. We undertook a pilot trial of computerised cognitive behavioural therapy (CCBT) for the treatment of depression in people with MS to test the feasibility of undertaking a full trial.

**Methods:**

Participants with a diagnosis of MS and clinical levels of depression were recruited through out-patient clinics and postal screening questionnaires at two UK centres and randomised to CCBT or usual care. Clinical outcomes included the Beck Depression Inventory (BDI-II) and Multiple Sclerosis Impact Scale (MSIS-29) at baseline, 8 and 21 weeks. Feasibility outcomes included: recruitment rate; reasons for refusal, withdrawal and dropout; feasibility and acceptability of the proposed outcome measures; sample size estimation and variation in and preferences for service delivery.

**Results:**

Twenty-four participants were recruited. The recruitment rate, calculated as the proportion of those invited to fill in a screening questionnaire who were consented into the trial, was 4.1%. Recruitment through out-patient clinics was somewhat slower than through screening questionnaire mail-out but the overall recruitment yield was similar. Of the 12 patients in the CCBT arm, 9 (75%) completed at least four, and 6 completed all 8 CCBT sessions. For completers, the median time (IQR) to complete all eight CCBT sessions was 15 (13 to 20) weeks. Participants expressed concern about the face validity of the Beck Depression Inventory II for the measurement of self-reported depression in people with MS. The MSIS-29 was the patient-reported outcome measure which participants felt best reflected their concerns. The estimated sample size for a full trial is between 180 and 390 participants. NHS partners were not delivering CCBT in community facilities and participants preferred to access CCBT at home, with no one expressing a preference for use of CCBT in an alternative location.

**Conclusions:**

A definitive trial, with a recruitment window of one year, would require the participation of around 13 MS centres. This number of centres could be reduced by expanding the eligibility criteria to include either other neurological conditions or people with more severe depression. The MSIS-29 should be used as a patient-important outcome measurement.

**Trial registration:**

ISRCTN: ISRCTN81846800

## Background

Multiple Sclerosis (MS) is a chronic immune mediated disease of the central nervous system which affects around 0.1% of Caucasians of north and central European ancestry [1]. MS is characterized by a variety of symptoms including visual impairment, limb weakness, sensory disturbance, balance and postural problems, sphincter dysfunction, cognitive impairments, pain and fatigue [2]. In the majority of patients, the illness runs an initial relapsing remitting (RRMS) course characterized by episodes of acute neurological dysfunction followed by full or partial recovery, usually culminating in a secondary progressive (SPMS) course during which disability progresses gradually with or without occasional relapses, minor remissions and plateaus [3]. Reports suggest that 50% of patients with MS experience major depression during their lifetime and up to 40% may have depression at any one time [4,5]. A Cochrane review, last updated in mid-2005, suggested there was some evidence that cognitive behavioural therapy (CBT) could be effective for the treatment of depression in people with MS [6].

The UK National Institute for Health and Clinical Excellence (NICE) recommends Cognitive Behavioural Therapy (CBT) as a treatment for mild to moderate depression, and there has been an increasing interest in its use to help people to remain in the workplace [7-9]. As therapist-led CBT is often inaccessible or prohibitively expensive, computerised CBT (CCBT) is recommended by NICE as part of a stepped-care model for the management of mild or moderate depression, typically delivered in a primary care setting [10,11]. NICE recommended one package in particular, Beating the Blues, for treatment of mild to moderate depression [10]. However, the management of mental health problems is often complicated by the co-presence of chronic physical illness, and treatments which have been validated in populations without chronic physical illness may not be appropriate or effective in such circumstances [12]. More recent NICE guidance recommends CCBT for the treatment of depression in people with chronic physical conditions whilst recommending that further randomised controlled trials of psychological interventions are undertaken for this population [13].

Because of the challenges inherent in evaluating complex interventions such as CCBT, the Medical Research Council's Complex Intervention Framework recommends a stepwise approach to evaluation, with pilot work preceding a full randomised controlled trial (RCT)[14]. The full study would be an RCT with economic evaluation alongside the trial to test the hypothesis that CCBT is clinically and cost-effective, compared to usual treatment, for the treatment of depression in people with MS. In a previous paper we reported qualitative research assessing the target group's views on the acceptability and appropriateness of CCBT [15]. In this paper we report the results of a pilot trial, designed to assess the feasibility of a research protocol for a multicentre trial and to estimate the variance of the treatment effect. The pilot trial did not attempt to provide evidence for the clinical effectiveness of CCBT for the treatment of depression in people with MS. Therefore, we have written up our findings in accordance with recommendations for CONSORT-modifications for reporting the results of pilot studies and pragmatic trials [16,17].

The objectives of this pilot trial were to:

• identify recruitment rates and test practicalities of recruitment;

• identify withdrawal and dropout rates during treatment phase and three month follow up phase to estimate dropout over longer term;

• identify reasons for refusal, withdrawal and dropout;

• test feasibility and acceptability of the proposed outcome measures, including the client service receipt inventory (a questionnaire developed for the collection of information on costs, service utilisation and related matters) required for a full economic evaluation;

• identify effect size and its associated variability at end of treatment in order to calculate an appropriate sample size for the full trial;

• identify variation in use or delivery of the intervention both at home and in an external setting;

• identify rate of preference for use of intervention at home or elsewhere

## Methods

### Participants and setting

We invited 582 people diagnosed with MS to screen for a study evaluating an intervention for low mood by completing a screening questionnaire including prognostic and eligibility criteria and a copy of the Beck Depression Inventory II-21 Item (BDI) [18]. All invitees had relapsing remitting or secondary progressive MS, according to the modified McDonald criteria [19]. Invitations were made between October 2008 and July 2009: face-to-face by consultants in the Sheffield Teaching Hospitals NHS Foundation Trust MS clinic (n = 288); by the Sheffield MS nurses visiting patients (n = 4); and, using ink-signed personalised letters mailed out from a neuropsychologist at Sheffield (n = 40) as well as from consultant neurologists at the Walton Centre for Neurology and Neurosurgery NHS Trust, Liverpool (n = 250). At Sheffield potential participants were identified from clinic attenders, MS nurse patient visits and from patients with MS from the caseload of the neuropsychologist. At the Walton Centre potential participants were identified solely from the MS register. The MS services in both sites are specific entities and not general neurology clinics.

Respondents completed a second BDI before a screening interview at which they were screened for eligibility by a clinical psychologist. Inclusion criteria were: age of 18 years or above; BDI score of at least 14 on two consecutive occasions and no treatment from psychologist, psychotherapist or psychiatrist within the last three months. That is, the sample was rated as depressed, using the BDI, but was not necessarily self-identifying and treatment-seeking. Exclusion criteria were: poor English language skills or cognitive function (score of less than 24 on Mini Mental State Examination [20]); BDI score of at least 29 on two consecutive occasions; active suicidal ideas; current or life-time diagnosis of psychosis, organic mental disorder or substance dependency; Kurtzke Expanded Disability Status Scale (EDSS) score of 8.5 or above [21]). The EDSS is a tool for quantifying MS-related disability. Our threshold for ineligibility was intended to exclude those who were restricted to bed for much of the day and had only limited use of their arms, thus effectively precluding use of a computer mouse and keyboard. The clinical psychologist assessed participants for major depressive disorder using the Mini-International Neuropsychiatric Interview (MINI) [22], but this was not an eligibility criterion.

Potential participants who were identified as having severe depression (BDI score of at least 29) or active suicidal ideas during the screening process were contacted by the study clinical psychologists and briefly assessed to understand whether the reported symptoms were typical and advised to contact their GP if deemed necessary. Once recruited to the study, active monitoring of Beck Depression Inventory responses was the responsibility of the study manager who contacted the PCT mental health lead on identifying responses indicating active suicidal ideation. Responses were dealt with according to the primary care trust's individual protocols for handling suicidal ideation.

### Interventions

After consent, we randomised participants either to CCBT using 'Beating the Blues'^® ^(Ultrasis Ltd) or to usual care (Treatment as Usual - TAU). A central web-based randomisation service delivered by the Sheffield Clinical Trials Research Unit was used after patient eligibility had been confirmed. All TAU arm participants were offered the opportunity of accessing the intervention at the end of the trial. The study statisticians and principal investigator remained blinded to the treatment allocation codes until after the final analysis. Beating the Blues^® ^consists of eight computer-interactive sessions, of approximately 50 minutes each in duration, designed to be taken weekly. Each session consists of a mix of cognitive and behavioural strategies, which the user customises to their individual problems. A client service receipt inventory was used at baseline, 8 weeks and 21 weeks to identify concomitant medication and service use in both arms: the research protocol did not manualise or restrict treatment as usual.

### Clinical outcomes

The primary clinical endpoint was the mean change in self-reported symptoms of depression as measured on the BDI. Specifically, we measured the mean change in scores between the two arms as measured at baseline, 8 and 21 weeks (13 weeks post intervention). Secondary clinical endpoints, measured at the same time points, were: (1) MS specific Quality of Life (QoL), measured by the Multiple Sclerosis Impact Scale 29-item (MSIS-29 [23]) questionnaire (physical and psychological components); general health related QoL, measured by the summary score for the Short Form-36 items (SF-36 [24]); overall improvement of depression severity and anxiety, measured by Patient Health Questionnaire-9 item (PHQ-9 [25]) and Generalised Anxiety Disorder 7-item (GAD-7 [26]) questionnaires respectively. A client service receipt inventory was used to capture concomitant use of health services and medication between follow-ups, to test collection of cost and activity data.

### Feasibility outcomes

The pre-specified primary outcome was the recruitment rate, calculated as the proportion of those invited to fill in a screening questionnaire who were consented into the trial. The practicalities of recruitment were assessed descriptively (see next paragraph). Refusal, withdrawal and dropout from the study protocol were recorded. The feasibility, acceptability and appropriateness of data collection strategies were assessed descriptively and through item response rates. Variation in the delivery of CCBT by Primary Care Trusts (PCTs) was documented, in terms of whether they made provision for use at home and the level of support. Rates of preference for use of the intervention at home and withdrawal from treatment were calculated as a percentage of those randomised.

A short semi-structured telephone interview was conducted with all study participants on completion of CCBT during which they were asked how they felt about the processes of recruitment and randomisation, the location of CCBT, their reasons for dropping out or not completing (where appropriate) and the appropriateness of the quantitative outcome measures used in the study. The methods for data collection and analysis of this qualitative component have been reported previously [15].

### Feasibility criteria

No criteria for evaluating the feasibility (in the sense of formal 'stop/go' criteria) of a definitive study were identified prospectively (see discussion).

### Sample size

We used a sample size of 12 per group (n = 24), on the basis of feasibility and precision of estimates to be used to design the main study [27].

### Statistical methods

Primary analysis was an Intention To Treat (ITT) analysis which analysed all 24 participants according to their randomised treatment assignment ignoring non compliance, protocol deviations and withdrawal. Statistical analysis was mainly descriptive [16] with outcome variability and patient response profiles analysed using summary measures at different time points. For this study, and in planning future studies it is most useful to present the absolute final values for individuals, rather than by how much individuals change from their initial baseline values as this allows comparison with population norms and other population groups. For these reasons we have presented the former rather than the latter as it provides the necessary information about both change and absolute values. Response rates on questionnaire items were high and last observation carried forward was used to impute the few missing items. Questionnaire response rates are given as a fraction of the total number of questions answered in a questionnaire among patients followed up on a specific visit relative to the total items. For example, the denominators for BDI and MSIS-29 are 21 and 29 questions respectively. Sample size calculation for a definitive trial was performed using an ANCOVA model, approximated standardised effect sizes corresponding to small, medium and large effect sizes, expected drop out rate, outcome variability and conservative correlation structure from this pilot study. All analysis was performed in Stata version 11.1.

### Ethical approval

This study received ethics approval from Northern and Yorkshire Research Ethics Committee.

## Results

### Recruitment rates and practicalities of recruitment

Our initial aim was to recruit 24 participants between 22 October 2008 and 31 January 2009 (101 days; 7.1 participants/month) with candidates identified by three neurologists working in a single weekly specialist MS clinic (Sheffield Teaching Hospitals NHS Trust). We reached our recruitment target on 30 July 2009 (in 281 days; 2.6 participants/month) after adding a second centre (The Walton Centre NHS Foundation Trust, Liverpool). From the 288 invitation packs given out by Sheffield neurologists in the MS clinic, forms were completed and returned by 63 (21.8%) candidates from which we randomised 13 in 281 days (4.5% recruitment yield), or 1.4 participants per month. From the 250 recruitment packs sent out by post from The Walton Centre, forms were completed and returned by 64 (25.6%) of recipients, from which 10 participants were randomised in 149 days (4.0% recruitment yield), or two participants per month. Sheffield MS Nurses approached four patients and a further participant was identified and recruited in this way. A neuropsychologist, based in Sheffield also wrote to 40 patients, but none of the respondents were recruited. In the telephone interviews, no participants expressed any concern about the processes of recruitment and randomisation.

### Participant characteristics

As this was a pilot study the sample size was small and by chance the random allocation of participants to the intervention and control groups resulted in imbalances between the groups at baseline with respect to gender, MS type and depression severity (Table 1).

**Table 1:** Participant baseline characteristics

Characteristic	Scoring	**TAU**^**3**^	**CCBT**^**4**^	Total
		(n = 12)	(n = 12)	(N = 24)
		n (%)	n (%)	n (%)
Gender	Male	5 (42%)	1 (8%)	6 (25%)
	Female	7 (58%)	11 (92%)	18 (75%)
Age (years)				
	Min to max	31 to 54	33 to 57	31 to 57
	Mean (SD)^1^	42 (7.0)	48 (7.7)	45 (7.9)
	Median (IQR)^2^	42 (37 to 47)	49.8 (42 to 55)	45 (39 to 51)
EDSS score				
	Min to max	0.0 to 6.0	2.0 to 6.5	0.0 to 6.5
	Mean (SD)	3.6 (1.8)	4.8 (1.7)	4.2 (1.8)
	Median (IQR)	3.5 (2.8 to 5.0)	5.5 (3.5 to 6.3)	4.0 (3.0 to 6.0)
Centre				
	Liverpool	5 (42%)	5 (42%)	10 (42%)
	Yorkshire	7 (58%)	7 (58%)	14 (58%)
MS type				
	Relapsing-remitting	12 (100%)	7 (58%)	19 (79%)
	Primary progressive	0 (0%)	5 (42%)	5 (21%)
BDI total score (0 weeks)				
	Min to max	15 to 29	16 to 27	15.0 to 29.0
	Mean (SD)	23 (5.2)	21 (4.0)	22 (4.7)
	Median (IQR)	25 (19 to 29)	22 (17 to 25)	22 (18 to 26)
				

^1 ^SD: standard deviation; ^2 ^IQR: inter-quartile range; ^3 ^TAU: Treatment as Usual; ^4 ^CCBT: Computerised Cognitive Behavioural Therapy.

### Refusal, withdrawal and dropout

**Figure 1:**
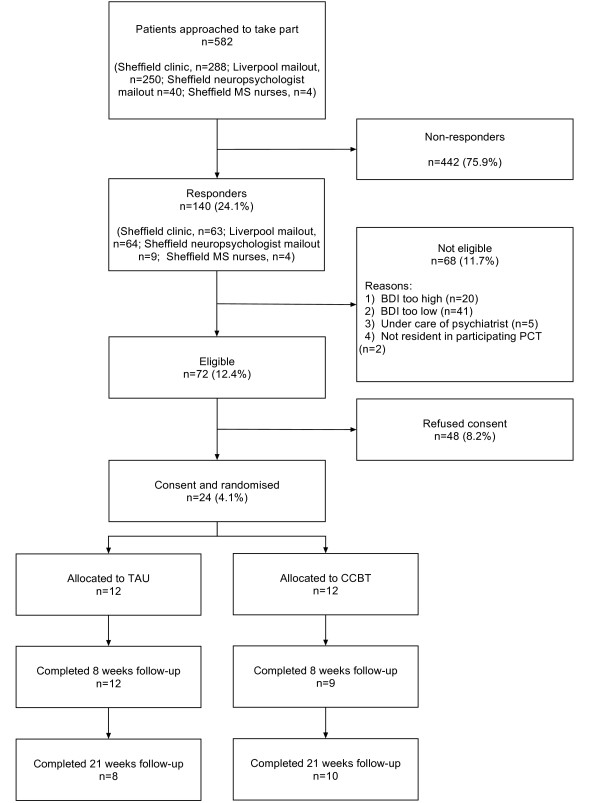
**CONSORT diagram**.

Of 582 patients invited to take part, 140 responded of whom 48 were not interested in participating, 68 were ineligible and 24 were randomised (Figure 1). Therefore the primary outcome, the recruitment rate, calculated as the proportion of those invited to fill in a screening questionnaire consented into the trial, was 4.1%. Of those who responded but were ineligible: 41 suffered from minimal depression (BDI of 13 or less); 20 were suffering from severe depression (BDI of 29 of more on two separate occasions); five were already seeing a psychiatrist or similar; and two were living in non-participating PCTs. Two participants in each arm were lost to follow-up at 21 weeks post-randomisation. No patients were formally withdrawn from the study.

### Feasibility, acceptability, appropriateness of data collection strategies

The collection of outcome data by postal questionnaire proved to be challenging, with multiple attempts to obtain data from many participants, particularly at later outcome assessment points. Nevertheless, primary outcome assessments were available for 21 (88%) participants at 8 weeks from randomisation (or end of treatment if later) and 18 (75%) participants at a further three months' follow-up. Furthermore, the completion rate for those questionnaires returned was high (Table 2).

**Table 2:** Questionnaire item response rates for available questionnaires

Questionnaire	Follow up Time point	Number of participants			TAU %		CCBT %		Overall %	
	
		All	TAU	CCBT	Min to max	median	Min to max	median	Min to max	median
BDI	0 weeks	24	12	12	95-100	100	100-100	100	95-100	100
	8 weeks	21	12	9	95-100	100	100-100	100	95-100	100
	21 weeks	18	8	10	91-100	100	71-100	100	71-100	100

MSIS-29	0 weeks	23	11	12	96-100	100	100-100	100	96-100	100
	8 weeks	20	12	8	96-100	100	100-100	100	96-100	100
	21 weeks	19	10	9	96-100	100	96-100	100	96-100	100

PHQ-9	0 weeks	23	11	12	90-100	100	100-100	100	90-100	100
	8 weeks	21	12	9	90-100	100	100-100	100	90-100	100
	21 weeks	18	8	10	90-100	100	100-100	100	90-100	100

GAD-7	0 weeks	23	11	12	71-100	100	100-100	100	71-100	100
	8 weeks	21	12	9	100-100	100	100-100	100	100-100	100
	21 weeks	18	9	9	100-100	100	100-100	100	100-100	100

SF-36	0 weeks	23	11	12	91-100	100	97-100	100	91-100	100
	8 weeks	21	12	9	66-100	100	100-100	100	66-100	100
	21 weeks	19	9	10	94-100	100	100-100	100	94-100	100

The patient reported outcome measure which the participants felt best reflected their concerns was the MSIS-29. During the telephone interviews, participants expressed concern about the face validity, for people with MS, of the questionnaires used for the measurement of self-reported depression, including the Beck Depression Inventory II. In particular they were concerned about the three symptoms, fatigue, sleep and concentration, which are also somatic symptoms of MS.

"[The questions are] not really appropriate for people with MS because they are things that you would have anyway. They're not actually concerned with depression like not having good sleep. I mean, that's a very common problem with MS is you don't have refreshing sleep." (ID 271)

No issues were identified with the client service receipt inventory designed for the collection of cost and activity data. Identification of prognostic variables, particularly the EDSS and MS Type, from patient records was not always possible and had to be obtained directly from participants' neurologists for the purposes of the study in some cases.

### Variability of outcome measures and sample size estimation

**Figure 2:**
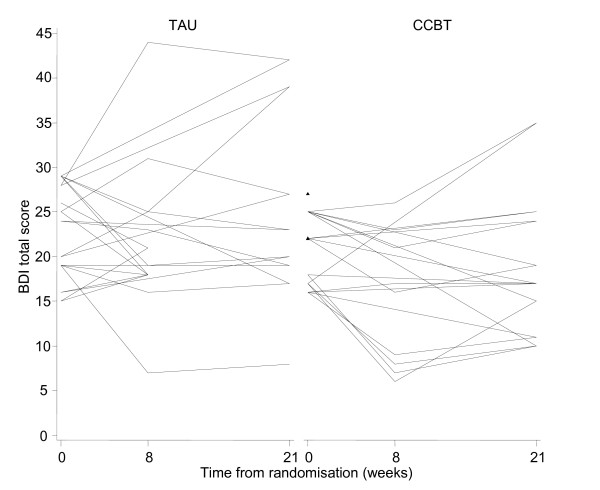
**BDI-II patient profiles (n = 24)**.

**Figure 3:**
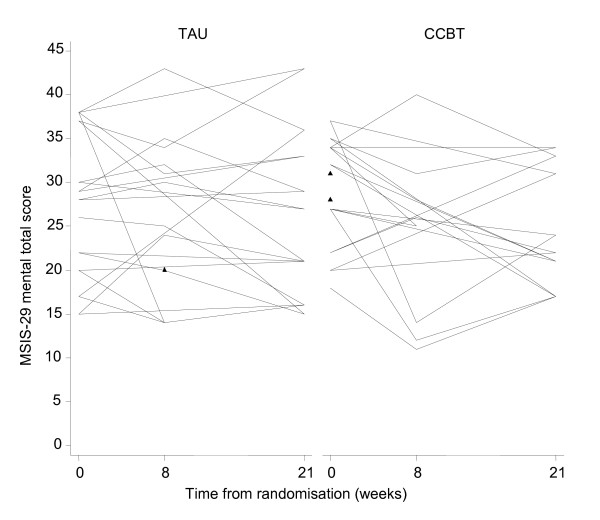
**MSIS-29 Patient Profiles (n = 24)**.

**Figure 4:**
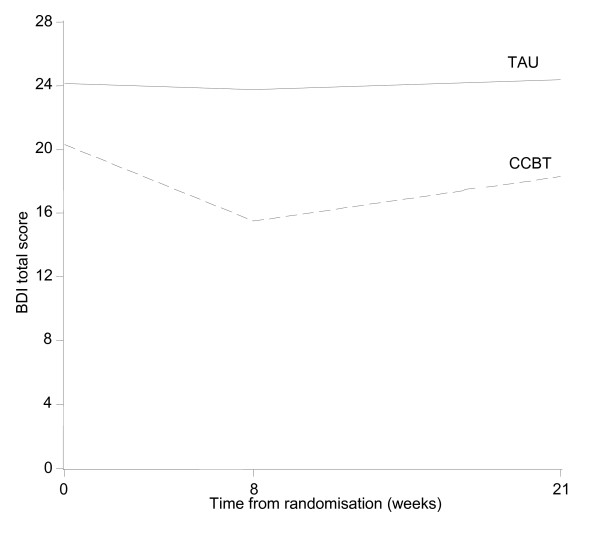
**BDI-II Mean profile (complete cases n = 18)**.

**Figure 5:**
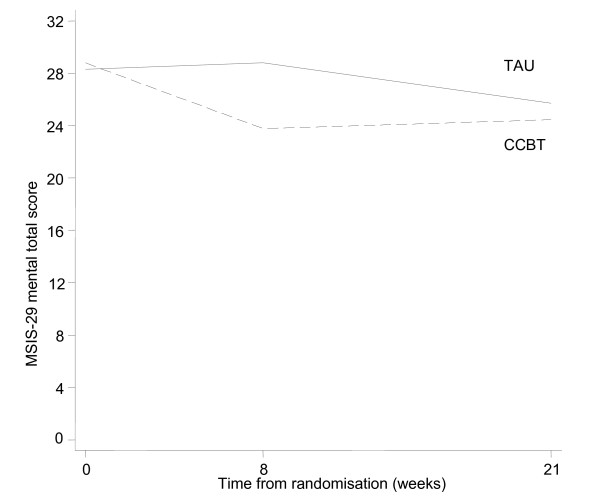
**MSIS-29 mean profile (complete cases n = 18)**.

Patient profiles with respect to clinical outcomes are reported in Table 3 and Figures 2, 3, 4 and 5. The usual approach to estimating sample size is to base it on an important clinically significant change. However, the difficulties of assessing a clinically significant change in BDI in the MS population have been reported previously with no available guideline [28]. An alternative approach is to base the effect size on the estimated mean change in BDI between the two groups. We decided against doing this as baseline imbalances with respect to gender and MS-type (Table 1) would produce bias in the estimated intervention effect.

**Table 3:** Summary measures of patient profiles for primary and secondary outcomes stratified by intervention group

Outcome Measure	Follow -up	TAU				CCBT			
BDI		n	Mean(SD)	Median(IQR)	Min-Max	n	Mean(SD)	Median(IQR)	Min-Max
		
	Baseline	12	23.3(5.2)	24.5(19.0-28.5)	15.0-29.0	12	21.0(4.0)	22.0(17.0-25.0)	16.0-27.0
	8 weeks	12	22.1(9.1)	20.0(18.0-25.0)	7.0-44.0	9	14.8(7.5)	16.0(8.0-21.0)	6.0-26.0
	21 weeks	8	24.4(11.4)	21.5(18.0-33.0)	8.0-42.0	10	18.3(7.9)	17.0(11.0-24.0)	10.0-35.0

MSIS-29 subscale									

Physical	Baseline	11	60.7(20.9	68.0(38.0-78.0)	32.0-94.0	12	62.8(14.2)	65.0(50.5-71.5)	42.0-87.0
	8 weeks	12	58.3(18.7)	60.5(42.5-74.0)	30.0-85.0	8	58.8(19.0)	65.5(40.0-71.0)	33.0-84.0
	21 weeks	10	60.8(22.0)	57.5(44.0-81.0)	31.0-97.0	9	55.8(18.0)	58.0(43.0-65.0)	27.0-82.0

Psychological	Baseline	11	27.3(8.2)	28.0(20.0-37.0)	15.0-38.0	12	28.8(6.2)	29.5(24.5-34.0)	18.0-37.0
	8 weeks	12	26.8(8.9)	27.5(20.0-33.0)	14.0-43.0	8	23.4(10.3	25.5(13.0-29.5)	11.0-40.0
	21 weeks	10	25.7(9.5)	24.0(16.0-33.0)	15.0-43.0	9	24.4(6.6)	22.0(21.0-31.0)	17.0-34.0

Instead we used approximate effect sizes expressed as a fraction of the pooled standard deviation of outcomes from this pilot which correspond to small, moderate and large standardised effect sizes. The standard deviations of the BDI, MSIS-29 psychological and physical domains were 8.3, 8.2 and 18.2 respectively, and variability of these outcome measures stratified by intervention group are shown in Table 4. Data at all time-points were included in these calculations and as such provide conservative estimates of the true standard deviation for these measures. The results related to the change in BDI and MSIS among complete cases are given in Table 5.

**Table 4:** Variability in outcomes (all time points) by intervention group

Outcome measure	Intervention group Mean (SD)		
	
	TAU	CCBT	All
BDI	24.1 (9.0)	18.0 (6.7)	20.7 (8.3)

MSIS-29 subscale			

Psychological	26.6 (8.6)	25.9 (7.7)	26.3 (8.2)

Physical	59.9 (19.9)	59.5 (16.5)	59.7 (18.2)

Mean and SD for BDI and MSIS-29 subscales based on data at all time-points

**Table 5:** Mean (SD) of change in primary and secondary outcomes relative to their baseline measurements.

Outcome measure	Follow-up	TAU		CCBT	
BDI		N	Mean (SD)	n	Mean (SD)
		
	8 weeks	12	-1.17 (8.1)	9	-5.33 (4.7)
	21 weeks	8	0.25 (8.8)	10	-2.00 (5.1)

MSIS-29 subscale					

Physical	8 weeks	11	-1.82 (7.8)	8	-7.00 (12.9)
	21 weeks	10	-2.20 (11.0)	9	-7.89 (12.3)

Psychological	8 weeks	11	0.18 (5.1)	8	-5.88 (9.5)
	21 weeks	10	-2.60 (5.6)	9	-4.33 (8.0)

A mean difference of change in 5 points on the BDI corresponds to a moderate standardised effect size and equates to the upper estimate of the likely effect size [29]. To have 90% power to detect this effect size (assuming correlation between follow-up measurements of 0.75, an attrition rate of 25% and a fixed type 1 error rate of 5%) a total of 70 participants would be required. A difference between groups of 3 or 2 points would correspond to the lower estimates of the likely effect size and this would require a total sample size of 180 and 390 respectively at 90% power. The summary of sample size estimates at varying levels of power and effect size (expressed as an absolute value and as equivalent standardised effect size) are given in Table 6.

**Table 6:** Sample size estimates for a definitive trial stratified by outcome measure, power and effect size

Outcome measure	Power (%)	Effect size		Sample Size per group	Total sample size (attrition adjusted)
				
		Approximate mean difference	Standardised		
BDI	90	5	0.6	35	70
		3	0.3	90	180
		2	0.2	195	390
	
	80	5	0.6	25	50
		3	0.3	65	130
		2	0.2	145	290

MSIS-29 subscale					
Psychological	90	3	0.3	95	190
		2	0.2	220	410
	
	80	3	0.3	70	140
		2	0.2	155	310
	
Physical	90	6	0.3	115	230
		4	0.2	260	520
	
	80	6	0.3	85	170
		4	0.2	195	390

### Delivery of CCBT (home/external) and variation in delivery and use

In the Sheffield region, Primary Care Trust (PCT) mental health care teams administered the CCBT, facilitating access and undertaking safety monitoring (PCTs were publicly funded organisations responsible for commissioning acute services for local populations). The CCBT service provided to trial participants was the routine service provided to all patients referred to the PCT mental health care teams. Only one out of the five participating PCTs around the Sheffield centre formally provided any kind of community facility for accessing CCBT; PCT staff warned that clients rarely used the facility and it was closed during the course of the study. Although four out of five participating PCTs were happy to arrange alternative provision for those who did not have Internet access at home, all our participants requested home use of CCBT. In the Liverpool region, a specialist neuropsychologist, based in the acute hospital facilitated access to and provided technical advice on CCBT, but did not provide additional therapeutic input. No provision other than home-use was offered or sought.

No PCT with whom we worked offered advice or support for defining problems on which to work or applying the CBT model to individual problems. The advice given was essentially technical, focusing on using the software. The PCTs delivering CCBT reported that they assumed that patients who were non-adherent and non-contactable beyond two weeks had discontinued treatment and made no further effort to contact them. Some but not all PCTs informed the patient's GP in such a situation. Preference for use at home amongst users in the study was universal with no-one expressing a preference for use of CCBT in an alternative location. Only one participant in the TAU arm took up the offer of access to CCBT at the end of the trial.

### Withdrawal from treatment

Of the 12 patients in the CCBT arm, 9 (75%) completed at least four CCBT sessions. This translated to a reasonably high compliance rate of CCBT although only six (50%) of the patients in this arm completed all intended eight CCBT sessions. Of these six patients, the median time (IQR) to complete all eight CCBT sessions was 15 (13 to 20) weeks against an intended eight weeks time frame. Only one person receiving CCBT formally requested discontinuation of treatment (after Session 6 of 8) citing time and lack of enthusiasm as reasons. Of the other non-completers, three also contributed qualitative data on their non-completion, one indicating lack of time, one that she no longer felt the need for treatment for her depression, and another citing computer hardware issues.

### Other concomitant service and medication use

Four participants received some kind of talking therapy outside of the research protocol during the trial, one in the CCBT arm and three in the TAU arm. Thirteen participants received anti-depressants during the trial: seven in the CCBT arm; six in the TAU arm.

## Discussion

Recruitment to this study was slower than expected. Dropouts and losses to follow-up were comparable with studies evaluating CCBT in non-MS populations: reported dropout rates for CCBT range from 0-75% (mean percentage dropout rate 32%, SD 16.52), which is comparable to dropout rates for other psychological therapies [30]. Data collection by postal questionnaire was challenging. Participants found problems with the face validity of the depression inventories which include somatic symptoms of depression which are also symptoms of MS (the BDI-II and the PHQ-9), despite the fact that one has been psychometrically validated previously for use in people with MS by several research teams [31,32]. The difficulties associated with assessing mood in people with physical illness is recognised and has been discussed previously [ 13,33]. However, the important point from this study is that participants expressed concerns about completing the measures and this may affect completion rates and validity in terms of consistency of participants' approach to responses throughout the course of the study. If a study is to evaluate impact on a patient-important outcome measure relevant to patients with MS then it should include the MSIS-29, which provides a measure of the physical and psychological impact of the condition from the patient's perspective [23]. This scale used on its own would reduce the ability to compare outcomes with other studies so it should be used in addition to general population scales such as the BDI, which has been used extensively in the general population.

A recent systematic Cochrane review identified a number of strategies proven to be effective in increasing response to postal questionnaires [34]. Fortunately, we had employed a few of these strategies including using a short, personalised (screening) questionnaire and including a second copy of the questionnaire at follow up. We also highlighted the University's involvement in the study. However, any future study could also employ other proven methods including using followup contact, use of stamped returned envelopes (as opposed to franked return envelopes), first class mailing and assurance of confidentiality in the letter of invitation.

Only a small percentage of people meeting the eligibility criteria for both MS and depression were successfully consented into the study. The population approached were not actively seeking treatment for mood-related symptoms and this may have been a contributing factor to the low consent rates. One other study has also identified that people with MS, while at higher risk of depression, are rarely treatment-seeking, at least in a primary care context [35].

A limitation of this study is that it did not prospectively define formal 'stop/go' criteria for a definitive study, allowing researchers to assess the main study as not feasible, feasible with protocol modifications, or feasible without modifications (with or without close ongoing monitoring of protocol feasibility and implementation). This was recommended as good practice for pilot studies by Thabane and colleagues subsequent to the finalisation of our protocol and our grant award [16].

This pilot trial has demonstrated that recruitment of the required sample size for a full trial from the relevant population would be challenging as the numbers of participants recruited per site were very low and a high proportion of the prevalent cases had been approached in the recruitment period. Consequently, extending the recruitment period at particular centres would result in diminishing returns in terms of participant recruitment. Postal screening via the Walton Centre resulted in 10 participants in 5 months, face to face recruitment at STH resulted in 14 participants in 9 months. From this evidence a full trial of 180 participants would require at least 13 sites, particularly considering that recruitment may be slower in a full-scale trial, where a team is reliant on other centres, than in a pilot study. This would require the participation of all the large, research active MS centres. Assuming 13 centres would be sufficient to recruit the required sample, the time to obtain all the necessary approvals and set up the sites would be considerable and 12 months at minimum should be allowed for this stage alone. In addition, time and resources should be allocated to allow for recruitment of additional sites if necessary

As we have tested recruitment using both identification of potential participants from an MS Register (Walton Centre) and through patient presentation at clinic (STH) either of these approaches could be utilised, so the service configuration would not be a limitation. However, the method of recruitment would also have to be considered carefully as we focused on a population who were screened for depression and not one which was actively seeking help for depression; this may have influenced the low uptake rate. In this regard, trialists may be in a difficult position: on the one hand, people with MS show unusually low levels of help-seeking for their depression, making recruitment through systematic mass screening more appealing [35]; on the other hand, psychotherapeutic studies in which patients are recruited through systematic screening rather than their routine caregiver show significantly lower effect sizes and the results of such a trial have poorer external validity [36]. Considering all these factors for a future full trial it may be better to extend the participant population to include people with other neurological conditions or even chronic conditions more widely. In addition, the eligibility criteria could be extended to include people with severe depression as in the ongoing Randomised Evaluation of the Effectiveness and Acceptability of Computerised Therapy (REEACT) Trial (ISRCTN 91947481). (Twenty respondents to our screening questionnaire, who might otherwise have been randomised, were excluded on the basis of experiencing severe depression at two measurement points). However, the sample size required to detect a difference may vary as our estimations are not based on this population.

It would be essential to maximise participant follow-up. A range of contact methods should be sought at the outset including mobile phone numbers and e-mail addresses. Sufficient resources would be required to ensure persistent followup of non-responders.

A further consideration for any future trial relates to the study design. The design of this study is described as CCBT vs TAU as the participants in the CCBT arm received CCBT in addition to any care, medication or services they would have received if they had not been part of the study. The TAU arm could be criticised as not reflecting usual treatment for depression but being a no-treatment arm (or TAU for MS) due to low levels of help-seeking for depression by people with MS [35]. An alternative approach would be to assess all participants for depression and to agree clinical management prior to randomisation. Those receiving TAU would therefore be offered appropriate treatment for depression, those in the treatment arm would receive CCBT in addition. This would also ensure that screened patients who do not meet the eligibility criteria on the basis of severity of depression would receive the appropriate clinical care.

The time taken for participants to complete the study intervention (13 to 20 weeks from randomisation) was longer than expected or than recommended in the product manual, due to the poor adherence of participants with the weekly schedule. Adherence to treatment programme timescales is difficult to guarantee where treatment is delivered by local NHS services and not delivered in ideal conditions by the trial team. Outcome assessment time-points of future studies should be fixed relative to the point of randomisation and not the projected end of the study intervention, as treatment programmes often over-run. This minimises discrepancies between the timepoints at which the treatment and control participants are followed up. Fixed timepoint outcome assessment should be supported by systems to minimise mis-timed outcome measurements, for instance automated follow-up reminders, pre-scheduled follow-up appointments and requesting alternative and preferred modes of contact (landline, mobile, text, e-mail).

Participants in our previous related research reported that they would value more support in defining their problems and goals [15]. Since this research began, Spek and colleagues have demonstrated that CCBT programmes are markedly more effective when delivered with therapist support. However, in the PCTs with whom we worked, therapist support was only available where issues remain unresolved after a complete course of CCBT, although more recently, through the 'Improving Access to Psychological Therapies' service, the support of a psychological wellbeing practitioner within primary care should be available within the stepped care model recommended by NICE [8]. Whether such support is in fact available is likely to vary between sites [7]. Any future research protocol would have to balance the desire to provide and evaluate an optimised intervention (CCBT with concurrent therapist support) against the likelihood of therapist-supported CCBT becoming the norm in the UK healthcare setting. In either case, study teams should report any standardisation or variation in the implementation of the intervention (for instance in the level of therapist support) between study sites in line with the CONSORT modification proposed by Zwarenstein and colleagues [17]. We would not recommend evaluating the delivery of CCBT in community facilities as our experience here has shown that many NHS partners are not delivering it in this way and participants overwhelmingly chose to access CCBT at home.

More successful models of delivering CCBT and implementing future interventional research might be through either (a) the secondary care multi-disciplinary neurology teams (see for example work by Moss-Morris and colleagues [37]) or (b) Psychological Wellbeing Practitioners based in primary care trained by clinical neuropsychologists in the issues they need to know to support CCBT use in patients with MS. The commercial company which produces Beating the Blues^® ^(Ultrasis) has indicated that it intends to address the issues of appropriateness and acceptability of Beating the Blues^® ^to people with MS identified in our previous work [14] in future product developments. The choice of CCBT to be used in any future full trial should take into consideration whether these issues have been addressed and the range of alternative CCBT packages which may be available in the future.

## Conclusions

The results of this pilot study demonstrate that, if the intended full trial of the clinical and cost-effectiveness of CCBT for depression in people with MS were to proceed, a number of amendments to the pilot trial protocol would be required. A definitive RCT, with a recruitment window of one year, would require the participation of around 13 UK MS centres. This number could be reduced by expanding the eligibility criteria to include either other neurological conditions or people with more severe depression. The design of the definitive study will need to consider whether to optimise the delivery of CCBT through the provision of therapist support depending on the likelihood of this being deliverable in routine practice. Participants should access CCBT at home in line with patient-preference and because NHS partners are not typically delivering it in community facilities. Finally, the MSIS-29 should be used as a patient-important outcome measurement.

## Competing interests

The authors declare that they have no competing interests.

## Authors' contributions

CC was the chief investigator and drafted the manuscript. CC and GP conceived of the study. CC, GP, JF, BS, AO, CI, AR and EK designed the study. DH was the study co-coordinator, conducted qualitative research interviews and helped to draft the manuscript. The trial management group were CC, DH, AO, CI, AR, LM and all, together with BS, helped to implement the study on a day-to-day basis. MD and JF were the study statisticians and contributed to the statistical analysis plan. MD undertook the statistical analysis and helped to draft the manuscript. LM provided a perspective on living with multiple sclerosis. AT conducted qualitative research interviews. All authors read, commented on and approved the final manuscript.
